# Simplified, Physically Motivated, and Broadly Applicable Range-Separation Tuning

**DOI:** 10.1021/acs.jpclett.5c01441

**Published:** 2025-08-04

**Authors:** Aditi Singh, Subrata Jana, Lucian A. Constantin, Fabio Della Sala, Prasanjit Samal, Szymon Śmiga

**Affiliations:** † Institute of Physics, Faculty of Physics, Astronomy and Informatics, Nicolaus Copernicus University, Grudziadzka 5, 87-100 Toruń, Poland; ‡ Institute of Physics, Faculty of Physics, Astronomy and Informatics, Nicolaus Copernicus University in Toruń, ul. Grudzia̧dzka 5, 87-100 Toruń, Poland; ¶ 312432 Institute for Microelectronics and Microsystems (CNR-IMM), 73100 Lecce, Italy; § Center for Biomolecular Nanotechnologies, Istituto Italiano di Tecnologia, 73010 Arnesano, LE, Italy; ∥ School of Physical Sciences, 193155 National Institute of Science Education and Research, An OCC of Homi Bhabha National Institute, Bhubaneswar 752050, India

## Abstract

Range-separated hybrid (RSH) functionals with “ionization energy” and/or “optimal tuning” of the screening parameter have proven to be among the most practical and accurate approaches for describing excited-state properties across a wide range of systems, including condensed matter. However, this method typically requires multiple self-consistent calculations and can become computationally expensive and unstable, particularly for extended systems. In this work, we propose a very simple and efficient alternative approach to determine the screening parameter for RSH functionals solely on the basis of the total electron density of the system and the compressibility sum rule of density functional theory (DFT). This effective screening parameter achieves remarkable accuracy, particularly for charge-transfer excitations, surpassing the performance of previously suggested alternatives. Because it relies on only the electron density, the proposed approach is physically transparent and highly practical to automate DFT calculations in large and complex systems, including bulk solids, where “tuning” is not possible.

## Graphical abstract

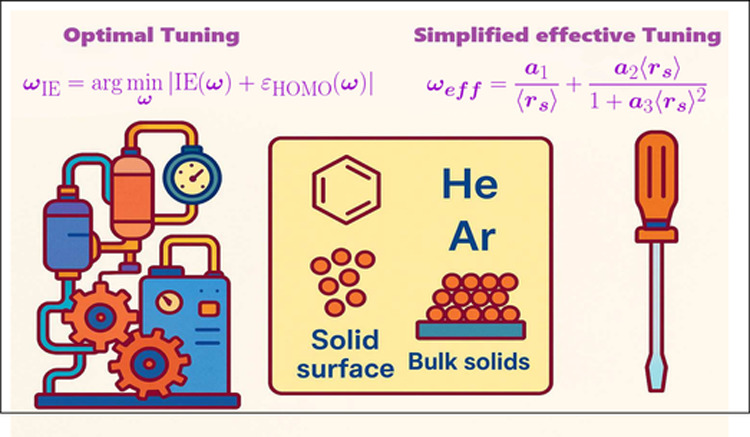

Since its advent, density functional theory (DFT)^1^ has become an indispensable formalism in interdisciplinary research, with significant applications in materials science and quantum chemistry.^1–4^ Ground-state properties can often be predicted with reasonable accuracy using cost-efficient semilocal approximations such as the local density approximation (LDA),^5^ generalized gradient approximations (GGAs),^6^ meta-GGAs,^7^ or a global hybrid^8^ level of approximation. In turn, the excited-state properties within time-dependent DFT (TD-DFT), particularly Rydberg and charge-transfer (CT) excitations, remain challenging.^9–19^ These limitations stem from the incorrect asymptotic decay of semilocal and global hybrid exchange-correlation potentials^20–22^ (critical for Rydberg states) and the lack of long-range exchange (essential for CT excitations),^23,24^ alongside the derivative discontinuity^25,26^ inherent in approximate density functionals. As an effective remedy, long-range corrected hybrid functionals with “ionization energy tuning” have been proposed by enforcing the exact, nonempirical Koopmans theorem (i.e., maintaining a constant for the long-range potential).^27–29^ Theoretically, the ionization potential-assisted tuning procedure optimizes range-separation parameter ω to enforce the exact ionization energy (IE) condition. The resulting value, ω_IE_, minimizes the expression^30,31^

1 $$\omega_{IE}=\arg\underset{\omega }{\min}\;\left|IE\left(\omega \right)+\varepsilon_{\mathrm{HOMO}}\left(\omega \right)\right|$$

Although this tuning procedure provides an enriching setting for small and medium-sized molecules,^32^ repeated ΔSCF calculations at the hybrid functional level become problematic. Consequently, it is very challenging to apply this scheme to periodic solids,^30^ solvated or embedded systems,^33^ systems with strong noncovalent interactions,^31^ large molecular chains, or nanostructured clusters.^34^ As a potential substitute, schemes such as effective charge-transfer distance tuning,^35^ global density-dependent (GDD) tuning,^36^ and electron localization function (ELF) tuning^37^ have been proposed (for solids, we also recall Wannier localization-based tuning^38,39^ ). These are one-shot (a black-box) strategies that circumvent the need for a laborious scan over ionization energies (IEs), making them exceptionally beneficial for larger molecular systems. However, these are not universal, and their applications for periodic solids have never been explored. For solids and clusters, several procedures for determining range-separated parameters have been developed that account for the distinct physical characteristics of extended systems.^40–42^ However, those approaches can generally not be transferred to finite or molecular systems, particularly for accurately predicting ionization potentials or fundamental gaps.^40,43,44^ Although all of these methods offer valuable insights, there remains a strong need for simple and physically transparent procedures, especially for broader applicability for “both-worlds” molecules and solid-state physics. Simplified yet accurate tuning protocols are still lacking.

Thus, as a significant advancement, this Letter introduces a simple yet elegant alternative approach for determining the range-splitting parameter in screened hybrid functionals. The proposed formalism is conceptually straightforward and highly versatile, making it applicable to a broad range of systems in both quantum chemistry and solid-state physics. Its generality and ease of implementation offer a promising route for improving the accuracy and efficiency of electronic structure calculations in diverse fields.

To establish the new formalism, we first recall the static density response function of the homogeneous electron gas (HEG), which can be conveniently represented as^45^

2 $$\chi \left(q\right)=\frac{\chi_{KS}\left(q\right)}{1-\left[v\left(q\right)+K_{\mathrm{xc}}\left(q\right)\right]\chi_{KS}\left(q\right)}$$

where χ_KS_(**q**) is the response in the Kohn-Sham (KS) framework, $v\left(q\right)=\frac{4\pi }{q^{2}}$ is the conventional Coulomb potential, and *K*_xc_(**q**) is the static XC kernel (all representation is in reciprocal space with reciprocal space vector **q** = **G** – **G**′) given by^46,47^

3 $$K_{xc}\left(q\right)=v\left(q\right)\left[\exp\left(-\frac{q^{2}}{4\omega^{2}}\right)-1\right]$$

where ω is the range-separation (or screening) parameter that distinguishes between the short- and long-range components of the electron–electron interaction. In range-separated hybrid (RSH) functionals, this separation is often introduced via an Ewald-like decomposition, i.e.

4 $$v\left(q\right)=\underset{\mathrm{SR}\;\mathrm{exchange}}{\underset{︸}{v\left(q\right)\left[1-\exp\left(-\frac{q^{2}}{4\omega^{2}}\right)\right]}}+\underset{\mathrm{LR}\;\mathrm{exchange}}{\underset{︸}{v\left(q\right)\;\exp\left(-\frac{q^{2}}{4\omega^{2}}\right)}}$$

Usually in the RSH functional, the ω is fixed as a constant (average value optimized for some reference data), a system-dependent constant (optimized for a given system), or even a position-dependent parameter. In the latter case, it was considered^48–51^

5 $$\omega ∼\frac{1}{r_{s}}+\frac{s}{r_{s}}+\frac{s^{2}}{r_{s}}+...$$

where $r_{s}=(\frac{3}{4\pi n\left(r\right)}{)}^{1/3}$ , $s=\frac{\left|\nabla n\left(r\right)\right|}{2k_{F}n\left(r\right)}$ , *k*_F_ = (3π^2^ *n*(**r**))^1/3^, and *n*(**r**) is the all electron total electron density. Hence, the Wigner–Seitz radius (*r*_s_) alone should be, in principle, sufficient to define the screening parameter of an RSH functional, which is the main focus of this paper and is further elaborated in the text. One may also argue that the density dependence of the range screening parameter was also proposed previously,^52^ but not established to use it in a practical way.^53^

Motivated by the above facts, we take a slightly different approach to construct ω. In particular, we consider the long-wavelength limit of eq 3, which is related to the exchange-correlation (XC) potential of the homogeneous electron gas (HEG) through the compressibility sum rule^54^

6 $$K_{xc}\left(q\to 0\right)=\frac{d^{2}}{dn^{2}}\left(n\epsilon_{xc}^{\mathrm{LSDA}}\left(r_{s},\zeta \right)\right)$$

where ϵ_xc_^LSDA^(*r*_s_, ζ) is the LSDA XC (PW91) energy per particle with ζ being the relative spin polarization. In *q* → 0, eqs 3 and 6 result in

7 $$\omega =\sqrt{-\frac{\pi }{K_{xc}\left(q\to 0\right)}}$$

This form is further simplified in ref 41,55,56 by considering a fit to the exact form,

8 $$\omega =\frac{a_{1}}{\left\langle r_{s}\right\rangle }+\frac{a_{2}\left\langle r_{s}\right\rangle }{1+a_{3}\langle r_{s}\rangle^{2}}$$

where *a*_1_ = 1.91718, *a*_2_ = −0.02817, and *a*_3_ = 0.14954. The local Seitz radius is given by $r_{s}={\left(\frac{3}{4\pi n}\right)}^{1/3}$ with *n* = (*n*_↑_ + *n*_↓_) and ⟨*r*_s_⟩ is the average over volume (unit cell). Although the calculation of ⟨*r*_s_⟩ is straightforward for bulk solids, tailored attention is required for finite systems, such as atoms and molecules, where the Seitz radius diverges in the tail of the density. To this end, for this kind of system, we consider another definition of the average *r*_s_

9 $$\left\langle r_{s}\right\rangle =\frac{\int w\left(r\right)r_{s}\left(r\right){\;d}^{3}r}{\int w\left(r\right){\;d}^{3}r}$$

The *w*(**r**) function is constructed in such a way as to catch the region where most of the electron density is localized (core and valence region). The same function is also used to define the volume for which we perform averaging of *r*_s_. Hence, we have defined this function as

10 $$w\left(r\right)=\mathrm{erf}\left(\frac{n\left(r\right)}{n_{c}}\right)$$

where erf is the error function with the cutoff density threshold (*n*_c_) defined as

11 $$n_{c}=\frac{n_{\mathrm{th}}}{\int n\left(r\right){\;d}^{3}r}$$

The cutoff density, *n*_c_, and corresponding radius, $r_{c}={\left(\frac{3}{4\pi n_{c}}\right)}^{1/3}$ , are dependent on system and size. Our threshold selection of *n*_th_ = 1.64 × 10^–2^ e/bohr^3^ provides consistent range-separation parameters (ω) for charge-transfer molecules through ionization energy (IE) tuning (see Tables SI5–SI7). While exact matching ω_eff_ ≃ ω_IE_ remains challenging due to their distinct physical origins, this *n*_th_ value represents a balanced choice for accurate charge-transfer excitation energies. Figure S1 further validates our approach, demonstrating close agreement between ω_eff_ and ω_IE_ for linear acenes (*n* = 2–40), poly­(*p*-phenylenevinylene) molecules [(PPV)_*n*=1–8_], and poly­(*p*-phenyl)­nitroaniline [O_2_N­(Ph)_*n*=1–11_NH_2_] oligomers. In contrast, alternative parameters (*n*_th_ = 10^–1^ e/bohr^3^ or *n*_th_ = 10^–3^ e/bohr^3^) yield significantly different ω_eff_ values.

Furthermore, within this scheme, integral ∫*n*(**r**′) d^3^ *r*′ effectively captures size-dependent variations and delocalization differences in linear molecular chains (discussed below). We also note that eq 10 tends to 1 when ∫*n*(**r**′) d^3^ *r*′ → ∞, recovering the bulk limit. It is crucial to acknowledge that this methodology, like other tuned range-separated hybrid approaches, inherits the fundamental challenge of size inconsistency, a limitation extensively documented by Karolewski et al.^34^ This deficiency arises directly from the density integral formulation in eq 11, which introduces a dependence on system size in parameter tuning. Thus, the present approach as well as GDD and the other tuned RSH functionals will fail to calculate the molecular properties where size consistency plays a key role.

To illustrate the novelty of the construction, we present a comparative plot of the Wigner–Seitz radius for the CN molecule (geometry from ref 57) and product *r*_s_(**r**)*w*(**r**) in the same panel in Figure 1. As demonstrated, the quantity *r*_s_(**r**)*w*(**r**) accurately reflects the behavior of *r*_s_(**r**) in regions of finite electron density, while it decays exponentially in the low-density tail regions. This exponential decay effectively captures the localization characteristics of the electron density, highlighting the ability of the constructed quantity to differentiate between the core and tail regions of the electronic distribution.

**Figure 1:**
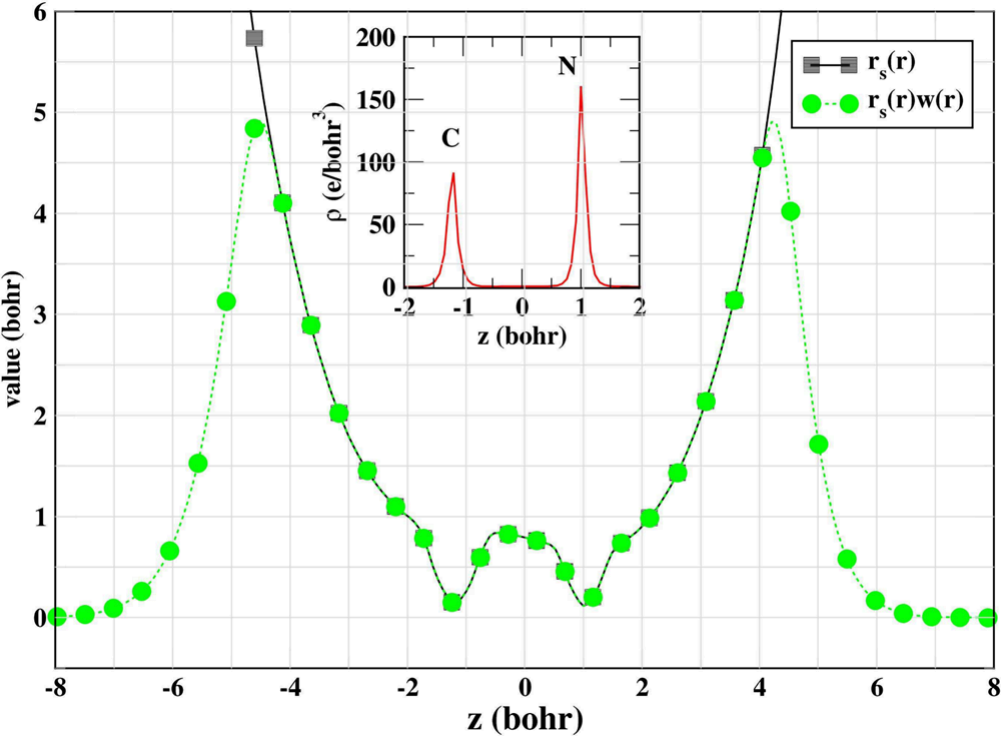
Electron density peaks for the CN molecule (inset) and the correct switching behavior of *r*_s_(**r**)*w*(**r**), which effectively suppresses the exponential growth of *r*_s_(*r*).

To demonstrate in practice the efficiency of this methodology, we performed all calculations using the long-range corrected hybrid functional LC-ωPBE, where range-separation parameter ω is defined according to eq 8. The computational tools employed include PySCF,^58^ NWChem,^59^ and Q-Chem.^60^ Specifically, PySCF is used to evaluate eq 9 and eq 8 according to the script deposited in the GitHub repository.^61^ The value of ω_eff_ does not need to be evaluated self-consistently. Instead, we recommend obtaining the electron density using the PBE exchange-correlation (XC) functional and then using this density to construct ω_eff_. This part of the calculation is carried out using the cc-pVDZ basis set. Importantly, we also observe that ω_eff_ varies only moderately with the choice of the XC functional and basis set, and such variations have a negligible effect on the final results. We note that a similar scheme is also employed in the evaluation of ω_GDD_.^62^ Moreover, NWChem and Q-Chem are utilized to perform all ground-state and TD-DFT calculations. The basis sets used in these calculations are specified either in the Supporting Information or within the caption of each table. The solid-state calculations are performed in the Vienna Ab initio Simulation Package (VASP).^63–66^

We first validate our approach by comparing the computed ionization energies of small atoms with available CCSD­(T) and experimental values,^67^ as shown in Figure 2. We refer to our newly tuned range-separated parameter as ω_eff_. The ionization energies computed from HOMO energies for the LC-ω_eff_PBE value show good agreement with both reference data sets (MAE = 0.33 eV) outperforming the fix (ω = 0.4), LC-ω_0.4_PBE variant (MAE = 0.61 eV). Overall, the results are well balanced, with most values closely aligned along the diagonal line. One can also note how the ω_eff_ values (reported in the first column of Table SI1) differentiate between the system varying in the range ω_eff_ ∈ (0.22–0.42).

**Figure 2:**
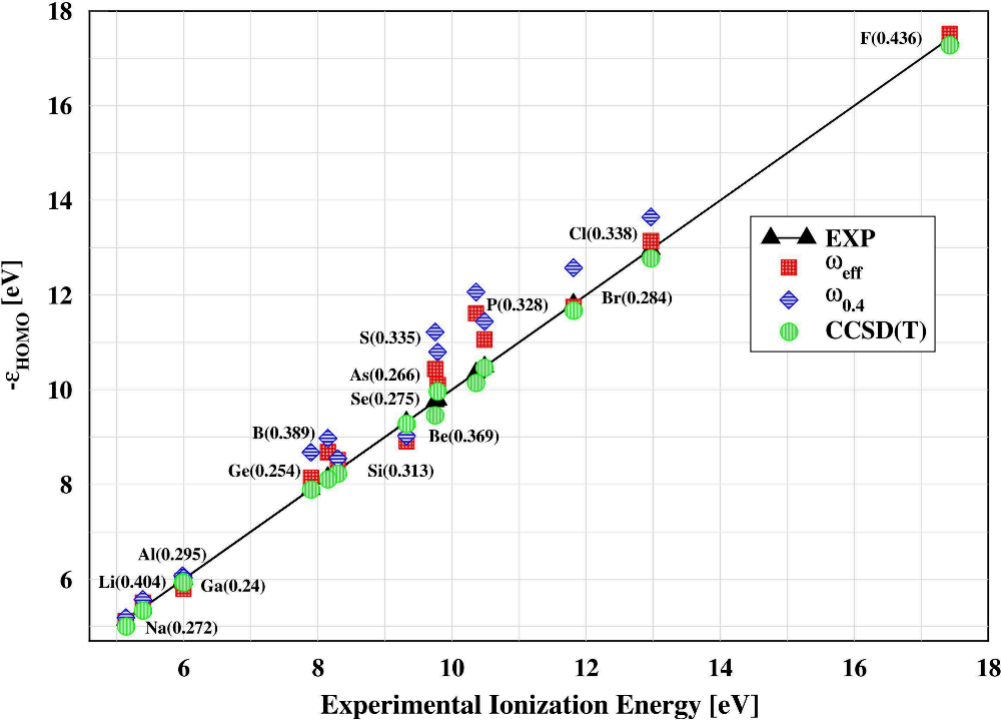
Ionization energies (IEs) of various open-shell atoms calculated using the LC-ω_*x*_PBE (*x* = 0.4, eff) functional. The results are benchmarked against experimental data^67^ and CCSD­(T) reference values.^67^ The values in parentheses correspond to ω_eff_ values. The aug-cc-pVTZ basis set^68^ was utilized in all calculations. The data are compiled in Table SI1.

Next, we compare the IE tuning strategy with the present approach for describing charge-transfer (CT) excitations within the TD-DFT framework. The computed excitation energies are summarized in Table 1 (the geometries were from ref 36), along with their corresponding range-separation parameters (ω values). Notably, the effective tuning parameter, ω_eff_, remains nearly constant across the set of studied molecules, a behavior also reported for the GDD approach in ref 36.

**Table 1:** TD-DFT Charge-Transfer Excitation Energies Calculated Using the LC-*ω*_*x*_PBE (*x* = IE, eff) Functional^a^

	ω (bohr^–1^)		LC-ωPBE (eV)		
molecule	ω_IE_	ω_eff_	ω_IE_	ω_eff_	*E*_ref_ (eV)
aminobenzonitrile	0.293	0.275	5.38	**5.36**	5.26
aniline	0.305	0.280	**5.85**	6.04	5.87
azulene	0.241	0.266	3.72	**3.91**	3.89
	0.241	0.266	**4.68**	**4.68**	4.55
benzonitrile	0.297	0.280	**6.69**	6.65	7.10
benzothiadiazole	0.443	0.269	**4.43**	4.47	4.37
dimethylaniline	0.266	0.266	**4.59**	**4.59**	4.47
	0.266	0.266	**5.44**	**5.44**	5.54
nitroaniline	0.284	0.274	4.44	**4.59**	4.57
nitrodimethylaniline	0.248	0.261	4.13	**4.35**	4.28
phthalazine	0.275	0.272	3.78	**3.82**	3.93
	0.275	0.272	4.35	**4.30**	4.34
quinoxaline	0.341	0.268	4.78	**4.74**	4.74
	0.341	0.268	**6.02**	6.13	5.75
	0.341	0.265	**6.43**	6.22	6.33
twisted DMABN	0.279	0.260	**3.81**	3.78	4.17
	0.279	0.260	5.17	**4.91**	4.84
dipeptide	0.325	0.264	7.99	**8.22**	8.15
β-dipeptide	0.296	0.258	8.00	**8.48**	8.51
	0.296	0.258	9.28	**8.79**	8.90
*N*-phenylpyrrole	0.464	0.261	5.72	**5.53**	5.53
	0.464	0.261	6.65	**6.15**	6.04
DMABN	0.257	0.263	**4.89**	5.02	4.94
					
MAE (eV)			0.22^a^	**0.12**	

a The ionization energy (IE)-tuned range-separation parameter, *ω*, and the excitation energies are from ref 36. The theoretically best estimated (TBE) values (last column) taken as reference excitation energies are from ref 69. All calculations employ the def2-TZVPD^70^ basis set. The best results are highlighted in bold.

b TBE used as described in ref 69 and MAE recalculated.

The excitation energies obtained using the LC-ω_eff_PBE functional show strong agreement with those from the IE-based tuning method, indicating that the ω_eff_ values effectively capture long-range electron–hole interactions characteristic for CT states.

Remarkable improvements are observed for certain systems when using the LC-ω_eff_PBE functional, particularly for nitrodimethylaniline, azulene, nitroaniline, twisted DMABN, dipeptide, and β-dipeptide, where excitation energies align more closely with reference values. Overall, our method demonstrates superior accuracy with a MAE of 0.12 eV, substantially outperforming the standard IE-tuning strategy, which exhibits a MAE of 0.22 eV. Additionally, employing ω_eff_ significantly enhances the prediction accuracy over the GDD-derived ω_GDD_ values, which display a larger MAE of 0.19 eV.^36^ Given that ω_0.3_ approximates the mean of ω_IE_ and ω_eff_, we provide LC-ω_0.3_PBE data in Table SI2 for reference. This functional similarly produces a 0.19 eV MAE, matching ω_GDD_’s performance. These findings highlight the ω_eff_ parameter’s predictability and scalability for forecasting CT excitations in a variety of molecular systems.

Figure 3 presents a comparative analysis of various methods for charge-transfer (CT) excitations across the benchmark test set listed in Table 1. As shown, the LC-ω_eff_PBE functional yields the lowest MAE of the different LC-ωPBE variants, demonstrating superior accuracy relative to those of several widely used traditional methods. In particular, LC-ω_eff_PBE outperforms B3LYP (MAE = 0.68 eV), PBE0 (MAE = 0.56 eV), CAM-B3LYP (MAE = 0.22 eV), and LRC-ωPBEH (MAE = 0.17 eV) in this context. We have also extensively compared our results with linear-response coupled cluster singles and doubles (LR-CCSD) results, which yield here a MAE of about 0.31 eV.^69^ It is readily apparent that the poorest options for capturing CT excitations would be PBE0 and B3LYP functionals, which can be related to their wrong asymptotic decay of exchange-correlation potential.^22^ Notably, despite LRC-ωPBEH^74–76^ being explicitly tailored for systems with charge-separation characteristics, the LC-ω_eff_PBE functional demonstrates superior performance, even surpassing it in accuracy. Thus, this indicates its robustness and reliability for modeling CT excitations. A comprehensive overview of the data is available in Table SI2.

**Figure 3:**
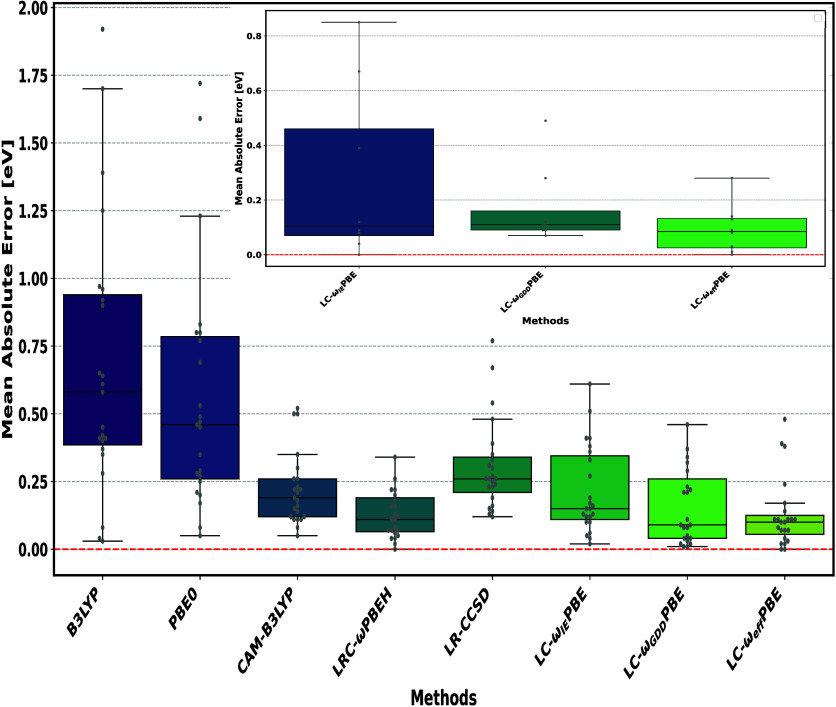
Box plots for the mean absolute error (MAE) of the benchmark test set of Table 1. The LC-ω_IE_PBE, B3LYP, PBE0, CAM-B3LYP, and LRC-ωPBEH data are from ref 36, whereas the LR-CCSD values are from ref 69. For Table 2, the box plot is given in the inset for the three methods. The data are available in Tables SI2 and SI3, respectively.

Next, the performance of the present method is benchmarked for singlet excitations of small open-shell molecules (the geometries are from ref 36), which are known to be challenging for the IE-tuning approach. As shown in Table 2, optimized ω_IE_ values cannot always be obtained. For example, no IE-tuned value could be found for the CN radical, where ω_IE_ → ∞.^36^ In contrast, the ω_eff_ values are more systematic and exhibit significantly less variation across different systems compared to IE tuning. For radicals such as OH and NCO, singlet excitation energies are generally overestimated by LC-ω_IE_PBE relative to LC-ω_eff_PBE. Overall, LC-ω_eff_PBE yields a MAE of 0.10 eV, which is substantially lower than that obtained from both ω_IE_ and ω_GDD_ methods.^36^ Notably, the IE and GDD approaches reveal significant changes in ω, consequently inflating the calculated singlet excitation energies. Crucially, LC-ω_eff_PBE not only demonstrates robust performance for closed-shell systems but also achieves accurate predictions for open-shell charge-transfer (CT) excitations, establishing its versatility across diverse systems.

**Table 2:** TD-DFT Singlet Excitation Energies within TDA Approximations for Several Open-Shell Molecules Calculated Using the LC-*ω*_*x*_PBE (*x* = IE, eff) Functional^a^

		ω (bohr^–1^)		LC-ωPBE (eV)		
molecule	transition	ω_IE_	ω_eff_	ω_IE_	ω_eff_	*E*_ref_ (eV)
BeF	°π	0.496	0.290	4.20	4.18	4.13
BH_2_	°B_1_	0.482	0.366	1.29	1.28	1.18
CN	°π	–	0.367	–	1.47	1.33
HCF	^1^A″	0.468	0.340	2.37	2.36	2.49
NH_2_	^2^A_1_	0.659	0.353	2.02	2.11	2.11
NO	^2^Σ^+^	0.600	0.382	5.74	6.08	6.12
OH	^2^Σ^+^	1.547	0.371	4.77	4.02	4.09
NCO	^2^Σ^+^	1.515	0.349	3.75	3.16	2.89
						
MAE (eV)				0.29^a^	0.10	

a The IE-tuned reference results are from ref 36. The theoretically best estimated values are from ref 71. All calculations employed the def2-TZVPD basis set.

b We consider as a reference the TBE values from ref 71 and recalculate MAE. Detailed error statistics available in Table SI3.

Next, we turn our attention to organic photovoltaic (OPV) materials. Optimized range-separated hybrid functionals have proven to be highly effective in reliably predicting the electronic properties of these materials, offering precision in modeling critical charge-transfer and excitation behaviors.^43^ These organic systems are typically large, which makes conventional optimal tuning (tuned using both *N* (HOMO) and *N* + 1 (LUMO) molecular orbital energies) procedures computationally expensive. In this context, the use of a simplified yet effective range-separation parameter such as ω_eff_ may offer a highly practical alternative without sacrificing accuracy.

For benchmarking purposes, we consider the same representative set of OPV molecules that were studied in ref 43. These geometries were obtained from ref 43. The performance of the LC-ω_eff_PBE functional is illustrated in Figure 4. An initial analysis of the ω_eff_ values reveals good agreement with optimal tuned values for most systems. Notable deviations can be seen, e.g., thiadiazole, PTCDA, or C_60_. However, these deviations have a minimal impact on the calculated electronic and excitonic properties. The HOMO energies computed with LC-ω_eff_PBE align closely with the reference diagonal, indicating accurate predictions. A similar trend is observed for the HOMO–LUMO gaps, with LC-ω_eff_PBE values showing excellent agreement with the benchmark *GW* results. Furthermore, the optical gaps calculated using LC-ω_eff_PBE match very closely with the optimally tuned Baer, Neuhauser, and Livshits (OT-BNL) values reported in ref 43, reaffirming the reliability of the present approach.

**Figure 4:**
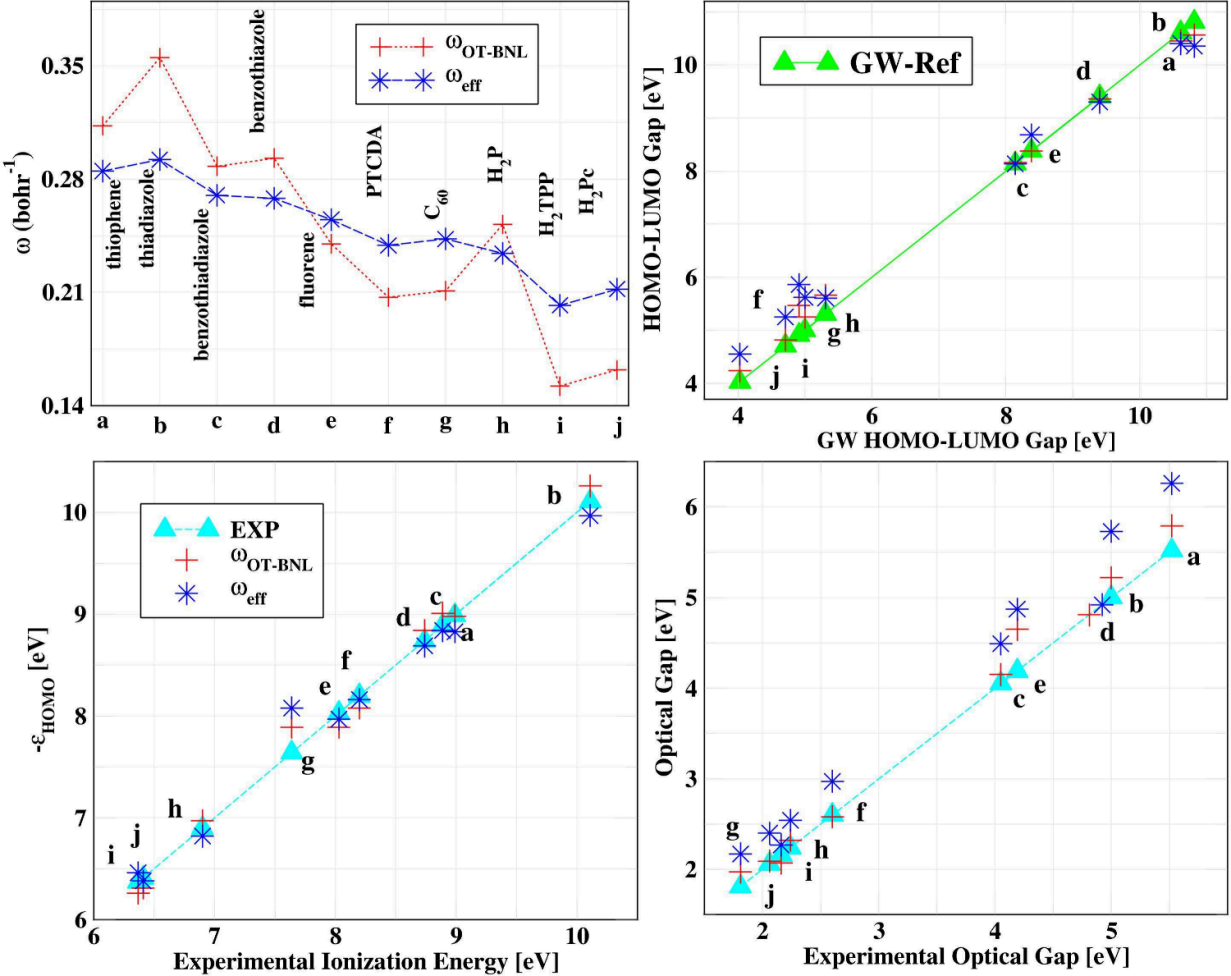
Variation of ω, HOMO energy, HOMO–LUMO gap, and optical gap of relevant organic photovoltaic (OPV) molecules. The LC-ω_OT‑BNL_PBE, *GW*, and experimental results are from ref 43. Calculations are performed using the cc-pVDZ basis set. Comprehensive data are available in Table SI4. Note that for LC-ω_OT‑BNL_PBE the screening parameter is optimized with respect to both *N* (HOMO) and *N* + 1 (LUMO) molecular orbitals.

Overall, these results highlight the effectiveness and practical applicability of LC-ω_eff_PBE for large OPV molecules, offering a computationally efficient yet accurate alternative to traditional IP- and/or gap-tuning methods.

In Table 3, we present a mean error analysis of the vertical excitation energies of linear acene systems (the geometries are from ref 36), benchmarked against experimental reference values.^72^ The excitation comprises two distinct transition states: L_a_, corresponding to the HOMO → LUMO transition, and L_b_, which involves either a HOMO → LUMO+1 or a HOMO–1 → LUMO transition. These states exhibit unique properties. The L_a_ state displays significant ionic character in its wave function, while the L_b_ state is predominantly covalent, resembling the ground state’s characteristics.^72^ Notably, the simplified approach employing ω_eff_ achieves a MAE of 0.189 eV, which is comparable to the MAE of 0.171 eV obtained using ω_IE_. These results demonstrate that the streamlined methodology preserves accuracy in describing electronically complex linear conjugated systems. Furthermore, as shown in Table SI7, the value of ω_eff_ lies between those of ω_GDD_ and ω_IE_, supporting the observed performance trends.

**Table 3:** Absolute Deviations from Experimental Values for Vertical Excitation Energies in Linear Acene Rings, Benchmarked against Experimental Data from ref 72, and Mean Absolute Errors (MAEs)^a^

			TD-LC-ω_*x*_PBE Error (eV)		
molecule	transition	experimental	ω_GDD_	ω_IE_	ω_eff_
naphthalene	^1^L_a_	4.66	0.09	0.01	0.02
naphthalene	^1^L_b_	4.13	0.45	0.40	0.40
anthracene	^1^L_a_	3.60	0.03	0.11	0.10
anthracene	^1^L_b_	3.64	0.41	0.30	0.36
tetracene	^1^L_a_	2.88	0.07	0.18	0.10
tetracene	^1^L_b_	3.39	0.34	0.18	0.30
pentacene	^1^L_a_	2.37	0.05	0.16	0.08
pentacene	^1^L_b_	3.12	0.41	0.21	0.37
hexacene	^1^L_a_	2.02	0.03	0.14	0.06
hexacene	^1^L_b_	2.87	0.16	0.02	0.10
					
MAE (eV)			0.204	0.171	0.189

a Results for the LC-*ω*_*x*_PBE (*x* = GDD, IE) are from ref 36. All computations employed the def2-TZVPD basis set.

One of the most important features of tuned RSH functionals is the size dependence of the range-separation parameter (ω) with respect to the number of repeat units in conjugated systems, such as polyenes and alkane chains. This characteristic has been extensively studied in previous works.^77,78^ In such systems, time-dependent density functional theory (TD-DFT) calculations employing either global hybrid functionals or fixed-ω RSH functionals often suffer from delocalization or localization errors, leading to inaccurate electronic and optical properties. These challenges become even more pronounced in extended systems and nanoscale materials, as discussed in ref 79.

To validate the performance and size-scaling behavior of our present approach, we examine three prototypical classes of linear conjugated molecules: (i) linear acenes with *n* = 2–40 benzene rings, (ii) poly­(*p*-phenylenevinylene) oligomers [(PPV)_*n*=1–8_], and (iii) poly­(*p*-phenyl)­nitroaniline chains [O_2_N­(Ph)_*n*=1–11_NH_2_]. These structures were previously analyzed in the context of the GDD approximation in ref 36 (the geometries are from ref 36).

As shown in Figure 5, the ω_eff_ values predicted by our method closely follow the trends observed for the values tuned with the ionization energy (ω_IE_) and are consistently lower than those obtained from the GDD approach (ω_GDD_). This is true across all three classes of molecules, suggesting that ω_eff_ has a distinct cutoff, which is introduced in eq 11 that captures the correct size dependence associated with electronic delocalization, to which it is related.

**Figure 5:**
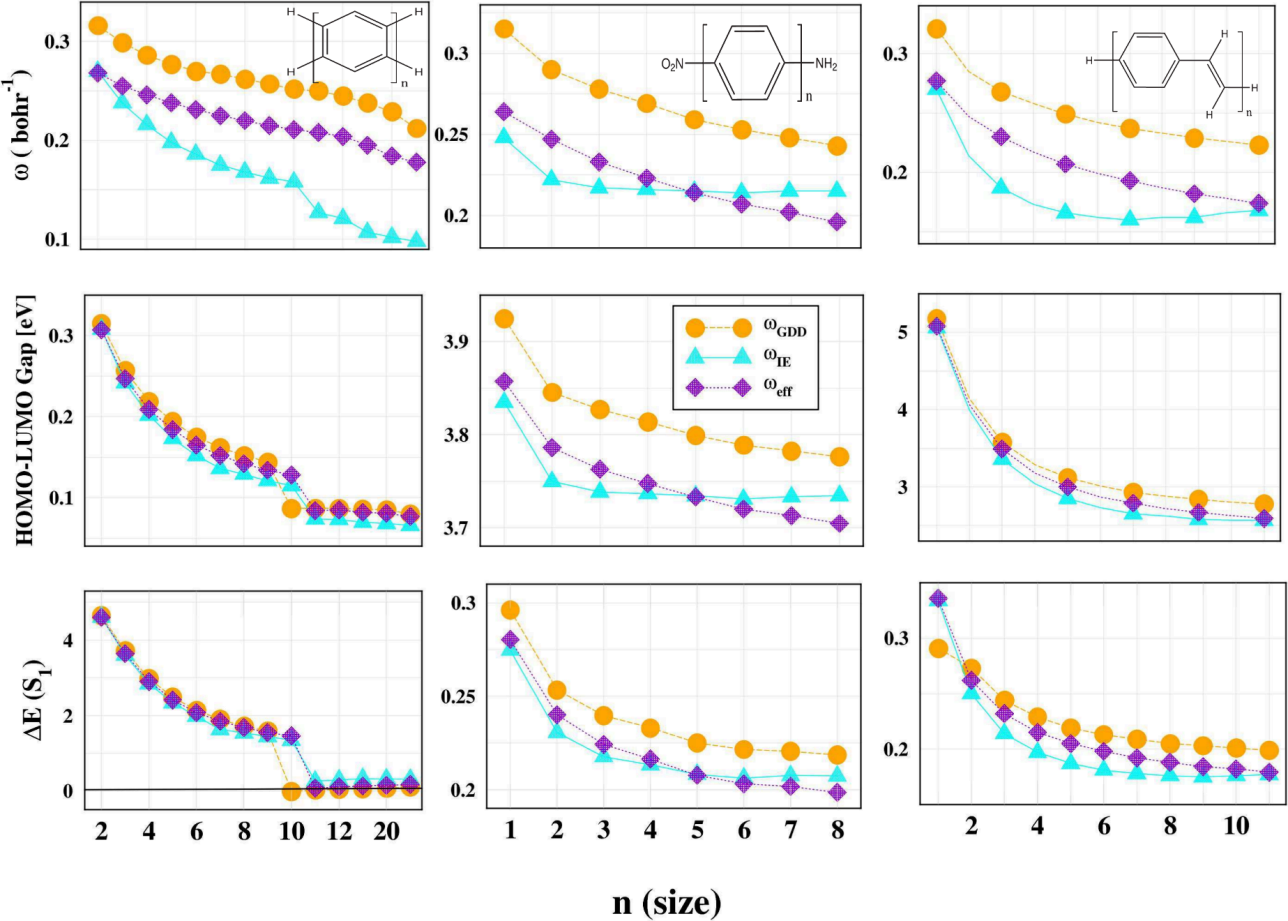
Range-separation parameters (ω), HOMO–LUMO gaps, and singlet excitation energies for linear acenes (*n* = 2–40), poly­(*p*-phenylenevinylene) molecules [(PPV)_*n*=1–8_], and poly­(*p*-phenyl)­nitroaniline oligomers [O_2_N­(Ph)_*n*=1–11_NH_2_] (from left to right, respectively). The ω_GDD_ and ω_IE_ values (along with their corresponding long-range corrected functional results) are from ref 36. All calculations were performed using the def2-ma-SVP basis set.^73^ Comprehensive data are available in Tables SI5–SI7.

In terms of electronic properties, both the HOMO–LUMO gaps and singlet excitation energies exhibit a monotonic decrease with an increase in chain length, which is in line with physical expectations. However, the results from LC-ω_IE_PBE show a more pronounced decay compared to LC-ω_GDD_PBE. The LC-ω_eff_PBE results, on the other hand, follow the trend of LC-ω_IE_PBE more closely, indicating that the present method better mitigates delocalization errors in long-chain systems. A particularly notable case arises for linear acenes with *n* = 10, where LC-ω_GDD_PBE predicts a negative excitation energy for one singlet state, an unphysical artifact that does not appear in either LC-ω_eff_PBE or LC-ω_IE_PBE, highlighting the improved stability of the ω_eff_-based approach. A discontinuity is evident when comparing acenes with *n* = 10 and *n* = 11 rings, which can be attributed to a sudden change in the KS gap. This observation implies the emergence of an open-shell biradicaloid singlet ground state in longer acenes.^36^ For the (PPV)_*n*=1–8_ oligomers, LC-ω_GDD_PBE significantly overestimates the HOMO–LUMO gap compared to both LC-ω_IE_PBE and LC-ω_eff_PBE, with the latter two in closer mutual agreement and better alignment with expected physical behavior. Similarly, for the poly­(*p*-phenyl)­nitroaniline chains, ω_eff_ again follows the size trend of ω_IE_ more accurately than does ω_GDD_. Although the differences in HOMO–LUMO gaps and excitation energies are less dramatic for this system, the consistency of the ω_eff_ trend supports the robustness of the present method. We also want to mention that for longer molecular chains, Wannier-optimized tuning may also be necessary.^39^

A natural question arises. Is the present formalism equally applicable to solid-state systems? To address this, we note that the proposed scheme can be effectively extended to bulk solids. In this context, the bulk-limit behavior of the screening parameter must be consistent with that described in ref 41. To achieve this, we recommend modifying eq 11 in a manner similar to that described in refs 83 and 84 using *n*_c_ = *n*_th_ = 6.96 × 10^–4^ e/bohr^3^, which corresponds to a Wigner–Seitz radius of *r*_c_ = *r*_th_ = 7 bohr, a suitable cutoff value for most bulk solids. However, in the case of solids, one must also account for dielectric-dependent effects^85^ in conjunction with range-separated screening.

To illustrate the applicability of this form of screening parameter, in Tables 4 and 5, we show the ω values for a few bulk solids and molecular crystals. Our test consists of (i) periodic bulk (geometries are from ref 41), (ii) 2D monolayers with various supercell heights or length perpendicular to the 2D layers (*c*) (geometries are from ref 86), (iii) surfaces (geometries are from ref 80), and (iv) molecular crystals (geometries are from ref 81). These are different kinds of solids that represent different physics of materials.

**Table 4:** Screening Parameters for Bulk and Monolayer Solids (all values in bohr^–1^)

material	μ^a^	μ_eff_^fit^ ^b^	μ_WS_ ^b^	μ_TF_ ^b^	ω_eff_
Periodic Bulk Solids					
Ar	0.74	0.54	0.52	0.56	0.51
C	0.90	1.24	0.76	0.68	1.25
Ge	0.62	0.79	0.45	0.52	0.72
Si	0.65	0.85	0.50	0.55	0.82
Periodic Monolayers					
graphene (*c* = 8 Å)	–	0.171	–	–	0.448
*h*BN (*c* = 20 Å)	–	0.072	–	–	0.369
*h*BN (*c* = 22 Å)	–	–	–	–	0.363
Surfaces^c^					
Si(111)-(2×1)	–	–	–	–	0.684
Ge(111)-(2×1)	–	–	–	–	0.674

a Values from ref 42.

b Values from ref 41.

c Structures are generated from ref 80.

**Table 5:** Screening Parameters, Band Gaps, and Positions of the Optical Transition from TD-DFT Calculations for Molecular Crystals^a^

					band gap			optical gap		
material	μ^a^	μ_TF_ ^b^	ω^OT‑SRSH^ ^c^	ω_eff_	*E*_g_ ^e^	*E*_g_ ^f^	*G*_0_*W*_0_@PBE^e^	TD-SRSH^e^	TD-SRSH(ω_eff_)	BSE^e^
NH_3_	0.53^d^	0.57^d^	0.375	0.599 (0.364)	7.9	8.3	7.7	7.1	7.8	7.1
CO_2_	–	–	0.405	0.546 (0.348)	11.2	11.8	11.2	10.7	11.1	10.8

a Values in parentheses are *ω*_eff_ values (in bohr^–1^) obtained from their respective gas phases. Here, all calculations of the SRSH functional are performed with the dielectric constant (*ϵ*) supplied in the Supporting Information of ref 81.

b From ref 42.

c From ref 41.

d Obtained from the gas phase of molecular crystals.^81^

e From ref 82.

f From ref 81.

g SRSH with ω_eff_.

For comparison between different screening parameters, we consider the following: (i) parameter μ fitted from the long-wavelength limit of the dielectric function,^42^ (ii) effective screening parameter μ_eff_^fit^ from ref 41, and (iii) μ estimated from the valence electron density (*n*_v_),^87^ defined as the number of valence electrons per unit volume. In ref 87, two forms of μ are proposed:

$$\mu_{\mathrm{WS}}={\left(\frac{4\pi n_{v}}{3}\right)}^{1/3}\;\mathrm{and}\;\mu_{\mathrm{TF}}={\left(\frac{3n_{v}}{\pi }\right)}^{1/6}$$

corresponding to the Wigner–Seitz (WS) and Thomas–Fermi (TF) screening models, respectively. Notably, the expressions for μ_eff_^fit^, μ_WS_, and μ_TF_ involve the unit cell volume (*V*_cell_) through *n*_v_ or in the expression itself. In contrast, effective frequency ω_eff_ proposed in this work differs in that contributions from regions of vanishing electron density are excluded. We argue that this approach is more general and applicable across a broader range of solid-state systems.

For bulk solids, the results closely match the μ_eff_^fit^ values proposed in ref 41, clearly indicating the practical applicability of the present form. For 2D monolayers, ω_eff_ is not changing much with respect to different vacuum sizes (*c*) (within periodic computational frameworks, “vacuum size” defines the engineered empty-space dimension isolating structures (surfaces, slabs, etc.), crucially governing boundary-condition implementations), which is very important as the present construction avoids the divergence of *r*_s_. For 2D monolayers, μ_TF_, μ_WS_, and μ_eff_^fit^ are not applicable as all of these expressions involve the volume of the unit cell and the volume depends on *c*. Thus, a larger *c* can give a larger volume, which makes μ_TF_, μ_WS_, and μ_eff_^fit^ not useful in this case. For example, the application of μ_eff_^fit^ for graphene and *h*BN monolayers results in very unphysical values, which also tend to zero. Thus, ω_eff_ is more general and robust (also remains an almost fixed value with different vacuum size). For the Si(111) and Ge(111) surfaces, we also obtained reasonable ω_eff_ values.

For molecular crystals, we also tested ω_eff_ against ω, tuned from the optimally tuned screened range-separated hybrid (OT-SRSH). As noted in Table 5, these values are slightly larger than those of the OT-SRSH.^81^ This is not surprising as ref 81 tuned the ω values from their respective gas phase, but not from bulk crystals. Table 5 also shows that ω_eff_ for the gas phase matches quite closely that of ω_OT‑SRSH_. Also, μ_eff_^fit^ matches closely μ and μ_TF_, indicating the versatility of this method. For fundamental or KS gaps, we obtain gaps of 8.3 eV for NH_3_ and 11.8 eV for CO_2_ molecular crystals, which are quite close to single-shot Green’s function-based method (*G*_0_ *W*_0_) values. For molecular crystals, no tuning is applied to their respective finite systems. Thus, these adjustments ensure that the method remains both robust and physically meaningful when applied to solid-state environments. Also, note that ω_eff_ values are quite close to μ_TF_ or obtained from fitting with RPA data.^82^ Finally, the obtained band gaps and positions of the first bright excitons are quite close to the benchmark TD-SRSH, *G*_0_ *W*_0_@PBE, and BSE for various excited-state properties.

Although this form is potentially insightful, particularly for extended systems with vacuum regions, such as two-dimensional materials, molecular crystals, or surfaces, this formulation (i.e., dielectric-dependent functional development) requires further investigation for practical use. It is worth noting that for bulk solids, the ΔSCF or IP-tuning or optimal-tuning procedures are generally not applicable because of the delocalization of orbitals;^44^ in this regard, the present method may offer advantages over the previously proposed methods.^29,31,82^

In conclusion, we have developed a simple and efficient single-shot approach to determine the range-separation parameter in long-range corrected hybrid functionals. The construction is based on a well-grounded, clear, and physically transparent framework. Unlike conventional tuned range-separated hybrid methods, this novel approach achieves remarkable accuracy with a significantly reduced computational cost across a wide variety of systems. This is important for modeling excitations in molecules. A key advantage of the present method is that the tuning behavior is derived solely from the electron density, making it easily transferable and broadly applicable. This represents a significant advancement toward achieving highly accurate results without the need for multiple tuning procedures. The application of ω_eff_ tuning demonstrates superior performance in the case of charge-transfer excitations, HOMO–LUMO gaps, and exciton energies. Overall, this development broadens the applicability of range-separated hybrid functionals and opens new possibilities for interdisciplinary research, including the starting point of high-level methods.^88^

In future work, we will explore the application of this methodology in ground-state DFT and linear-response TD-DFT calculations as well as its impact on functional, orbital, and density errors.^89^

## Setup for VASP Calculations

VASP calculations to evaluate ω_eff_ for periodic bulk solids are performed using the final electron density obtained from self-consistent calculations with the PBE exchange-correlation functional. A Γ-centered 15 × 15 × 15 **k**-point mesh and an energy cutoff of 600 eV are used. For periodic monolayers and surface systems, a Γ-centered 15 × 15 × 1 **k**-point grid is employed, with the same energy cutoff of 600 eV. In the case of molecular crystals, a Γ-centered 8 × 8 × 8 **k**-point mesh and a higher energy cutoff of 800 eV are used to evaluate ω_eff_ (based on the PBE density) and to perform TD-SRSH calculations.

## Supplementary Material





## Data Availability

All data supporting the findings of this study are available in the main text and the Supporting Information. The PySCF-based code for calculations of ω_eff_ values used in this work is publicly available in the repository.^61^

## Notes

Tables with raw data and additional figures that support the analysis (PDF)
Transparent Peer Review report available (PDF)
