# Supplementary material for: Simplified, Physically Motivated, and Broadly Applicable Range-Separation Tuning
Source: J Phys Chem Lett. 2025 Aug 4;16(32):8198–208. doi: 10.1021/acs.jpclett.5c01441 (PMC12359113; doi:10.1021/acs.jpclett.5c01441)
Supplement: Supplementary file 1 [file jz5c01441_si_001.pdf]

# Supporting Information for
## Simplified, Physically Motivated, and Broadly Applicable Range-Separation Tuning

Aditi Singh[a], Subrata Jana[a], Lucian A. Constantin[b], Fabio Della Sala[b,c], Prasanjit Samal[d], and Szymon Śmiga[a]

[a]) Institute of Physics, Faculty of Physics, Astronomy and Informatics, Nicolaus Copernicus University, Grudziadzka 5, 87-100 Toruń, Poland
[b]) Institute for Microelectronics and Microsystems (CNR-IMM), 73100 Lecce, Italy
[c]) Center for Biomolecular Nanotechnologies, Istituto Italiano di Tecnologia, 73010 Arnesano, LE, Italy
[d]) School of Physical Sciences, National Institute of Science Education and Research, An OCC of Homi Bhabha National Institute, Bhubaneswar 752050, India

July 8, 2025

# Contents

- References
- Figure
- Tables

# References


[1] Sivan Refaely-Abramson, Roi Baer, and Leeor Kronik PHYSICAL REVIEW B 84, 075144, 2011

[2] Epifanovsky, Evgeny and Gilbert, Andrew T. B. and Feng, Xintian and Lee, Joonho, The Journal of Chemical Physics, 155, 8, 084801, 2021

[3] Stein, Tamar and Kronik, Leeor and Baer, Roi, Journal of the American Chemical Society, 131, 8, 2818-2820, 2009

[4] Mandal, Aniket and Herbert, John M., The Journal of Physical Chemistry Letters, 16, 10, 2672-2680, 2025

[5] Aprá, E. and Bylaska, E. J. and de Jong, W. A. and Govind, N, The Journal of Chemical Physics, 152, 18, 184102, 2020

[6] Linstrom, Peter J. and Mallard, William G., Journal of Chemical & Engineering Data, 46,5, 1059-1063, 2001

[7] Loos, Pierre-François and Comin, Massimiliano and Blase, Xavier and Jacquemin, Denis, Journal of Chemical Theory and Computation, 17, 6, 3666-3686, 2021

[8] Pierre-François Loos, Anthony Scemama, Martial Boggio-Pasqua and Denis Jacquemin, Journal of Chemical Theory and Computation, 16, 6, 3720-3736, 2020

[9] Grimme, Stefan and Parac, Maja, ChemPhysChem, 4, 3, 292-295, 2003

# 1 Supplementary Figure

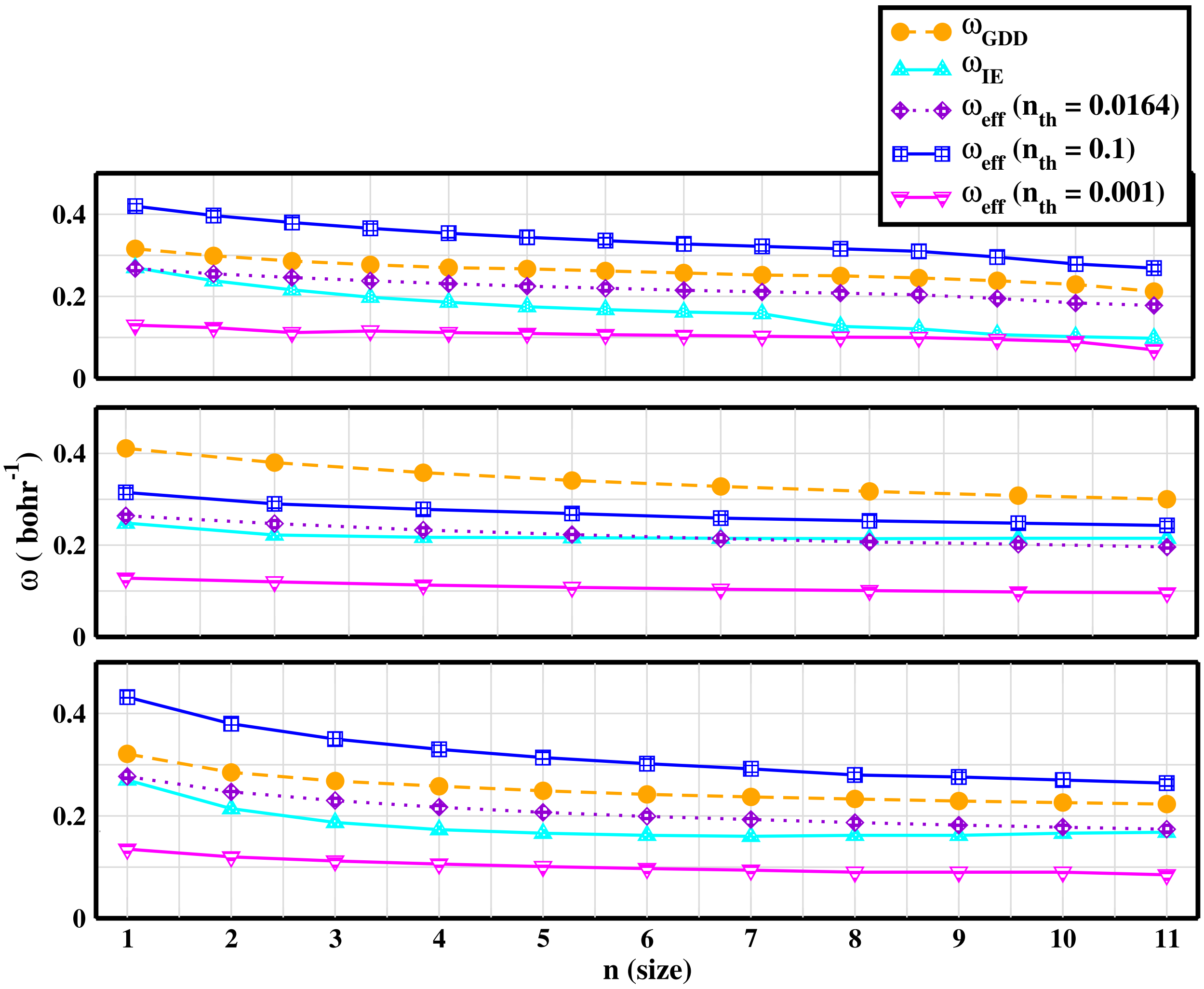


**Figure S1:** Range-separation parameter ($\omega$), for (from top to bottom): linear acenes ($n$ = 2-40), poly(p-phenylenevinylene) molecules [$(PPV)_{n=1-8}$], and poly(p-phenyl)nitroaniline [$O_2N(Ph)_{n=1-11}NH_2$] oligomers. The $\omega_{GDD}$ and $\omega_{IE}$ values are taken from Ref.[4] The $\omega_{eff}$ are calculated based on different values of $n_{th}$.

## 2 Supplementary Tables

**Table SI1:** Table shows Atomic Ionisation Potential (IP) computed as IP=$-\epsilon_{HOMO}$ (in eV) from different methods taken from Ref.[6], and calculations performed with LC-$\omega_{eff}$PBE functional using NWChem Ref.[5]. All calculations employ the aug-cc-pVTZ basis set.

| Atoms | $\omega_{eff}$(bohr$^{-1}$) | IP(CCSD(T)) | IP$^{expt}$ | IP(LC-$\omega_{eff}$PBE) | IP(LC-$\omega_{0.4}$PBE) |
|---|---|---|---|---|---|
| Li | 0.261 | 5.343 | 5.392 | 5.392 | 5.570 |
| Be | 0.346 | 9.285 | 9.323 | 8.914 | 9.023 |
| B | 0.369 | 8.233 | 8.298 | 8.517 | 8.547 |
| F | 0.423 | 17.275 | 17.423 | 17.513 | 17.279 |
| Na | 0.223 | 5.003 | 5.139 | 5.118 | 5.187 |
| Al | 0.278 | 5.938 | 5.985 | 5.967 | 6.071 |
| Si | 0.298 | 8.110 | 8.152 | 8.683 | 8.974 |
| P | 0.316 | 10.469 | 10.487 | 11.059 | 11.442 |
| S | 0.324 | 10.150 | 10.360 | 11.614 | 12.062 |
| Cl | 0.328 | 12.775 | 12.968 | 13.138 | 13.647 |
| Ga | 0.227 | 5.929 | 5.999 | 5.796 | 6.033 |
| Ge | 0.244 | 7.898 | 7.899 | 8.142 | 8.678 |
| As | 0.259 | 9.971 | 9.789 | 10.101 | 10.798 |
| Se | 0.268 | 9.460 | 9.752 | 10.438 | 11.219 |
| Br | 0.277 | 11.671 | 11.814 | 11.744 | 12.572 |
| MAE(eV) | | 0.11 | 0.00 | 0.33 | 0.61 |

**Note:** FIG. 2 graphically displays these data. Mean Absolute error (MAE) calculated with respect to experimental.

**Table SI2:** Table presents absolute error values for excitation energy (in eV). The theoretically best estimated (TBE) values are taken as reference from Ref. [7], while other values are from Ref. [4] and LR-CCSD from Ref. [7]. Calculations for LC-$\omega_{eff}$PBE are performed in NWChem. All calculations employ def2-TZVPD basis set.

| | | | | | | | | | | LC-$\omega$PBE | | | |
|---|---|---|---|---|---|---|---|---|---|---|---|---|---|
| Molecule | State | Symmetry | MO-combination | $E_{ref}$ | B3LYP | PBE0 | CAM-B3LYP | LRC-$\omega$PBEH | LR-CCSD | $\omega_{IE}$ | $\omega_{GDD}$ | $\omega_{eff}$ | $\omega_{0.3}$ |
| Aminobenzonitrile | $S_2$ | $A_1$ | 31-32 | 5.26 | 0.35 | 0.25 | 0.15 | 0.22 | 0.15 | 0.12 | 0.09 | 0.10 | 0.13 |
| Aniline | $S_3$ | $A_1$ | 25-29, 24-26 | 5.87 | 0.45 | 0.35 | 0.30 | 0.22 | 0.12 | 0.02 | 0.21 | 0.16 | 0.26 |
| Azulene | $S_2$ | $A_1$ | 34-36,33-35 | 3.89 | 0.28 | 0.21 | 0.16 | 0.06 | 0.13 | 0.17 | 0.11 | 0.02 | 0.06 |
| Azulene | $S_3$ | $B_2$ | 33-36,32-35 | 4.55 | 0.08 | 0.17 | 0.22 | 0.08 | 0.27 | 0.13 | 0.21 | 0.13 | 0.19 |
| Benzonitrile | $S_5$ | $A_2$ | 25-28 | 7.10 | 0.90 | 0.80 | 0.50 | 0.00 | 0.23 | 0.41 | 0.34 | 0.45 | 0.40 |
| Benzothiadiazole | $S_2$ | $B_2$ | 35-36 | 4.37 | 0.58 | 0.47 | 0.26 | 0.16 | 0.26 | 0.06 | 0.08 | 0.10 | 0.17 |
| Dimethylaniline | $S_1$ | $B_2$ | 33-35 | 4.47 | 0.04 | 0.05 | 0.22 | 0.07 | 0.19 | 0.12 | 0.20 | 0.12 | 0.30 |
| Dimethylaniline | $S_2$ | $A_1$ | 33-36 | 5.54 | 0.42 | 0.20 | 0.08 | 0.05 | 0.14 | 0.10 | 0.04 | 0.10 | 0.45 |
| Nitroaniline | $S_4$ | $A_1$ | 36-37 | 4.57 | 0.64 | 0.49 | 0.21 | 0.04 | 0.23 | 0.13 | 0.08 | 0.02 | 0.09 |
| Nitrodimethylaniline | $S_2$ | $A_1$ | 44-45 | 4.28 | 0.61 | 0.46 | 0.14 | 0.16 | 0.25 | 0.15 | 0.02 | 0.07 | 0.18 |
| Phthalazine | $S_1$ | $A_2$ | 34-35,34-39 | 3.93 | 0.41 | 0.28 | 0.12 | 0.11 | 0.33 | 0.15 | 0.01 | 0.11 | 0.00 |
| Phthalazine | $S_2$ | $B_1$ | 34-36 | 4.34 | 0.40 | 0.29 | 0.05 | 0.04 | 0.30 | 0.41 | 0.46 | 0.04 | 0.05 |
| Quinoxaline | $S_3$ | $B_2$ | 34-35 | 4.74 | 0.65 | 0.53 | 0.23 | 0.11 | 0.26 | 0.04 | 0.04 | 0.00 | 0.04 |
| Quinoxaline | $S_4$ | $A_1$ | 34-36 | 5.75 | 0.03 | 0.08 | 0.18 | 0.07 | 0.26 | 0.27 | 0.22 | 0.38 | 0.45 |
| Quinoxaline | $S_8$ | $B_1$ | 33-38 | 6.33 | 0.41 | 0.45 | 0.11 | 0.07 | 0.54 | 0.10 | 0.05 | 0.11 | 0.09 |
| Twisted DMABN | $S_1$ | $A_2$ | 39-40 | 4.17 | 0.96 | 0.83 | 0.19 | 0.12 | 0.24 | 0.36 | 0.23 | 0.39 | 0.24 |
| Twisted DMABN | $S_3$ | $B_1$ | 38-41 | 4.84 | 0.97 | 0.80 | 0.11 | 0.02 | 0.35 | 0.33 | 0.37 | 0.07 | 0.13 |
| Dipeptide[a] | $S_{12}$ | A” | 33-39,33-41 | 8.15 | 1.92 | 1.59 | 0.35 | 0.34 | 0.77 | 0.16 | 0.32 | 0.07 | 0.35 |
| $\beta$-dipeptide[a] | $S_{15}$ | A’ | 38-43 | 8.51 | 1.25 | 0.69 | 0.50 | 1.31 | 0.39 | 0.51 | 0.01 | 0.03 | 0.05 |
| $\beta$-dipeptide[a] | $S_{17}$ | A” | 37-49,37-43 | 8.90 | 1.70 | 1.72 | 0.52 | 0.20 | 0.67 | 0.38 | 0.85 | 0.11 | 0.17 |
| N-phenylpyrrole[a] | $S_1$ | $B_2$ | 37-39 | 5.53 | 0.92 | 0.77 | 0.26 | 0.13 | 0.31 | 0.19 | 0.09 | 0.00 | 0.16 |
| N-phenylpyrrole[a] | $S_4$ | $A_1$ | 37-40 | 6.04 | 1.39 | 1.23 | 0.12 | 0.18 | 0.48 | 0.61 | 0.29 | 0.11 | 0.20 |
| DMABN[a] | $S_2$ | $A_1$ | 39-40 | 4.94 | 0.37 | 0.27 | 0.11 | 0.26 | 0.16 | 0.05 | 0.03 | 0.08 | 0.16 |
| MAE(eV) | | | | | 0.68 | 0.56 | 0.22 | 0.17 | 0.31 | 0.22 | 0.19 | 0.12 | 0.19 |

a) These data have been recalculated from Ref. [4] using Ref. [7] TBE as reference. FIG. 3 graphically displays these data. MAE calculated with respect to $E_{ref}$.

**Table SI3:** Table presents absolute error values for excitation energy (in eV), where theoretically best estimated ($E_{ref}$) values are taken from Ref. [8]. Other values are taken from Ref.[4]. LC-$\omega_{eff}$PBE calculations are performed in NWChem. All calculations employ the def2-TZVPD basis set.

| **Molecule** | $\mathbf{E_{ref}}$ | **LC-$\omega_{IE}$PBE** | **LC-$\omega_{eff}$PBE** | **LC-$\omega_{GDD}$PBE** |
|---|---|---|---|---|
| BeF | 4.13 | 0.07 | 0.05 | 0.07 |
| BH2 | 1.18 | 0.11 | 0.10 | 0.11 |
| CN | 1.33 | – | 0.14 | 0.28 |
| HCF | 2.49 | 0.13 | 0.13 | 0.09 |
| NH2 | 2.11 | 0.09 | 0.00 | 0.09 |
| NO | 6.12 | 0.11 | 0.04 | 0.11 |
| OH | 4.09 | 0.68 | 0.07 | 0.12 |
| NCO | 2.89 | 0.86 | 0.27 | 0.49 |
| MAE(eV) | | 0.29 | 0.10 | 0.17 |

FIG. 3 graphically displays these data. MAE calculated with respect to $E_{ref}$

**Table SI4:** Table shows comparative analysis for HOMO energies, HOMO-LUMO gap, and optical gaps of relevant organic photo-voltaic (OPV) molecules (in eV) for $\omega_x$ (x=OT-BNL and eff) with reference data for OT-BNL, Experimental and GW from Ref. [1]. LC-$\omega_{eff}$PBE calculations are performed in Q-Chem Ref. [2]. All calculations employ the cc-pVDZ basis set. For LC-$\omega_{eff}$PBE we report also in the bracketes the values obtained in the def2-TZVPD basis set.

| **Molecule** | $\omega$ (bohr$^{-1}$) | | IP (eV) | | | HOMO-LUMO gap (eV) | | | 1st Singlet Excitation (eV) | | |
|---|---|---|---|---|---|---|---|---|---|---|---|
| | $\omega_{OT-BNL}$ | $\omega_{eff}$ | Exp | $\omega_{OT-BNL}$ | $\omega_{eff}$ | GW | $\omega_{OT-BNL}$ | $\omega_{eff}$ | Exp | $\omega_{OT-BNL}$ | $\omega_{eff}$ |
| (a)thiophene | 0.313 | 0.285 | 8.89 | 9.01 | 8.84 (8.97) | 10.61 | 10.45 | 10.40 (10.22) | 5.52 | 5.79 | 6.26 (6.03) |
| (b)thiadiazole | 0.355 | 0.292 | 10.11 | 10.26 | 9.97 (10.08) | 10.81 | 10.56 | 10.35 (10.08) | 5.00 | 5.22 | 5.73 (5.52) |
| (c)benzothiadiazole | 0.288 | 0.270 | 8.99 | 8.98 | 8.83 (9.01) | 8.14 | 8.16 | 8.14 (8.06) | 4.05 | 4.15 | 4.49 (4.35) |
| (d)benzothiazole | 0.293 | 0.268 | 8.74 | 8.84 | 8.69 (8.82) | 9.40 | 9.36 | 9.30 (9.19) | – | 4.81 | 4.92 (4.84) |
| (e)fluorene | 0.240 | 0.255 | 8.03 | 7.89 | 7.97 (8.08) | 8.38 | 8.38 | 8.68 (8.57) | 4.19 | 4.65 | 4.87 (4.75) |
| (f)PTCDA | 0.207 | 0.239 | 8.20 | 8.08 | 8.16 (8.34) | 5.00 | 5.25 | 5.62 (5.57) | 2.60 | 2.58 | 2.97 (2.88) |
| (g)$C_{60}$ | 0.211 | 0.243 | 7.64 | 7.89 | 8.08 (8.14) | 4.91 | 5.47 | 5.86 (5.82) | 2.24 | 2.32 | 2.54 (2.51) |
| (h)$H_2P$ | 0.252 | 0.234 | 6.90 | 6.97 | 6.82 (6.98) | 5.31 | 5.66 | 5.61 (5.60) | 2.16 | 2.07 | 2.27 (2.46) |
| (I)$H_2TPP$ | 0.152 | 0.202 | 6.37 | 6.26 | 6.46 (6.62) | 4.71 | 4.82 | 5.25 (5.24) | 2.06 | 2.09 | 2.40 (2.37) |
| (j)$H_2Pc$ | 0.162 | 0.212 | 6.41 | 6.31 | 6.38 (6.48) | 4.03 | 4.24 | 4.55 (4.50) | 1.81 | 1.97 | 2.17 (2.12) |
| MAE(eV) | | | 0.00 | 0.17 | 0.12 (0.13) | 0.00 | 0.20 | 0.40 (0.44) | 0.00 | 0.16 | 0.45 (0.37) |

**Note:** FIG. 4 graphically displays these data. MAE calculated with respect to the experiment and GW values.

**Table SI5:** Table presents singlet excitation energies and Kohn-Sham gap for linear acenes computed using optimally tuned LC-$\omega_X$PBE with (x=GDD,IE) from Ref. [4] and and LC-$\omega_{eff}$PBE calculated in Q-Chem, with TD-DFT (TDA). All calculations employ the def2-ma-SVP basis set.

| No. of | GDD | | | IE | | | eff | | |
|---|---|---|---|---|---|---|---|---|---|
| Rings | $\omega_{GDD}$ (bohr$^{-1}$) | $\Delta E(^1\mathrm{L}_a)$ (eV) | KS gap ($\mathrm{E}_h$) | $\omega_{IE}$ (bohr$^{-1}$) | $\Delta E(^1\mathrm{L}_a)$ (eV) | KS gap ($\mathrm{E}_h$) | $\omega_{eff}$ (bohr$^{-1}$) | $\Delta E(^1\mathrm{L}_a)$ (eV) | KS gap ($\mathrm{E}_h$) |
| 2 | 0.316 | 4.65 | 0.315 | 0.270 | 4.61 | 0.308 | 0.266 | 4.60 | 0.307 |
| 3 | 0.299 | 3.72 | 0.257 | 0.238 | 3.60 | 0.242 | 0.253 | 3.64 | 0.247 |
| 4 | 0.286 | 2.98 | 0.219 | 0.216 | 2.84 | 0.202 | 0.242 | 2.90 | 0.209 |
| 5 | 0.277 | 2.49 | 0.194 | 0.198 | 2.34 | 0.173 | 0.234 | 2.41 | 0.184 |
| 6 | 0.270 | 2.14 | 0.175 | 0.186 | 1.98 | 0.152 | 0.227 | 2.08 | 0.165 |
| 7 | 0.267 | 1.90 | 0.162 | 0.175 | 1.63 | 0.136 | 0.221 | 1.85 | 0.152 |
| 8 | 0.262 | 1.72 | 0.152 | 0.168 | 1.54 | 0.129 | 0.216 | 1.68 | 0.142 |
| 9 | 0.257 | 1.59 | 0.144 | 0.162 | 1.44 | 0.121 | 0.211 | 1.55 | 0.134 |
| 10 | 0.252 | -0.01 | 0.087 | 0.158 | 1.34 | 0.115 | 0.207 | 1.46 | 0.128 |
| 11 | 0.250 | 0.02 | 0.087 | 0.127 | 0.26 | 0.074 | 0.203 | 0.08 | 0.084 |
| 12 | 0.245 | 0.05 | 0.087 | 0.121 | 0.29 | 0.073 | 0.200 | 0.11 | 0.085 |
| 15 | 0.238 | 0.06 | 0.086 | 0.107 | 0.33 | 0.070 | 0.191 | 0.13 | 0.082 |
| 20 | 0.229 | 0.07 | 0.085 | 0.102 | 0.32 | 0.068 | 0.180 | 0.15 | 0.081 |
| 40 | 0.212 | 0.11 | 0.080 | 0.098 | 0.31 | 0.066 | 0.178 | 0.17 | 0.077 |

**Note:** FIG. 6 graphically displays these data.

**Table SI6:** Table presents singlet excitation energies and Kohn-Sham gap for $O_2N(Ph)_nNH_2$ oligomers computed using optimally tuned LC-$\omega_X$PBE with (x=GDD, IE) from Ref. [4] and and LC-$\omega_{eff}$PBE calculated in Q-Chem, with TD-DFT(TDA). All calculations employ the def2-ma-SVP basis set.

| $n$ | $\omega_{GDD}$ (bohr$^{-1}$) | $\Delta E(S_1)$ (eV) | KS gap($E_h$) | $\omega_{IE}$ (bohr$^{-1}$) | $\Delta E(S_1)$ (eV) | KS gap($E_h$) | $\omega_{eff}$ (bohr$^{-1}$) | $\Delta E(S_1)$ (eV) | KS gap ($E_h$) |
|---|---|---|---|---|---|---|---|---|---|
| 1 | 0.315 | 3.9248 | 0.2962 | 0.248 | 3.8349 | 0.2747 | 0.261 | 3.8573 | 0.2804 |
| 2 | 0.290 | 3.8454 | 0.2535 | 0.222 | 3.7498 | 0.2307 | 0.243 | 3.7860 | 0.2401 |
| 3 | 0.278 | 3.8271 | 0.2398 | 0.217 | 3.7385 | 0.2177 | 0.229 | 3.7630 | 0.2242 |
| 4 | 0.269 | 3.8139 | 0.2332 | 0.216 | 3.7367 | 0.2134 | 0.219 | 3.7475 | 0.2165 |
| 5 | 0.259 | 3.7992 | 0.2251 | 0.215 | 3.7344 | 0.2083 | 0.210 | 3.7331 | 0.2078 |
| 6 | 0.253 | 3.7887 | 0.2216 | 0.214 | 3.7308 | 0.2063 | 0.203 | 3.7202 | 0.2032 |
| 7 | 0.248 | 3.7825 | 0.2207 | 0.215 | 3.7333 | 0.2077 | 0.198 | 3.7130 | 0.2018 |
| 8 | 0.243 | 3.7764 | 0.2187 | 0.215 | 3.7345 | 0.2074 | 0.193 | 3.7046 | 0.1986 |

**Note:** FIG. 6 graphically displays these data.

**Table SI7:** The table presents singlet excitation energies and Kohn-Sham gap for $PPV_n$ computed using optimally tuned LC-$\omega_X$PBE with (x=GDD, IE) from Ref. [4] and LC-$\omega_{eff}$PBE calculated in Q-Chem, with TD-DFT (TDA). All calculations employ the def2-ma-SVP basis set.

| $n$ | $\omega_{GDD}$ (bohr$^{-1}$) | $\Delta E(S_1)^{\mathrm{b}}$ (eV) | KS gap ($E_h$) | $\omega_{IE}$ (bohr$^{-1}$) | $\Delta E(S_1)^{\mathrm{b}}$ (eV) | KS gap ($E_h$) | $\omega_{eff}$ (bohr$^{-1}$) | $\Delta E(S_1)^{\mathrm{b}}$ (eV) | KS gap ($E_h$) |
|---|---|---|---|---|---|---|---|---|---|
| 1 | 0.321 | 5.18 | 0.291 | 0.270 | 5.06 | 0.334 | 0.274 | 5.08 | 0.336 |
| 2 | 0.285 | 4.14 | 0.273 | 0.214 | 3.99 | 0.250 | 0.243 | 4.06 | 0.262 |
| 3 | 0.268 | 3.58 | 0.244 | 0.187 | 3.36 | 0.214 | 0.225 | 3.49 | 0.232 |
| 4 | 0.258 | 3.29 | 0.228 | 0.173 | 3.04 | 0.197 | 0.212 | 3.18 | 0.215 |
| 5 | 0.249 | 3.12 | 0.216 | 0.166 | 2.85 | 0.187 | 0.202 | 3.00 | 0.205 |
| 6 | 0.242 | 3.01 | 0.213 | 0.162 | 2.73 | 0.181 | 0.194 | 2.87 | 0.198 |
| 7 | 0.237 | 2.93 | 0.209 | 0.160 | 2.65 | 0.178 | 0.188 | 2.79 | 0.192 |
| 8 | 0.233 | 2.88 | 0.183 | 0.162 | 2.62 | 0.176 | 0.186 | 2.72 | 0.188 |
| 9 | 0.229 | 2.84 | 0.203 | 0.162 | 2.58 | 0.175 | 0.179 | 2.67 | 0.184 |
| 10 | 0.226 | 2.80 | 0.201 | 0.166 | 2.57 | 0.176 | 0.174 | 2.63 | 0.182 |
| 11 | 0.223 | 2.78 | 0.199 | 0.168 | 2.57 | 0.177 | 0.170 | 2.59 | 0.179 |

**Note:** FIG. 6 graphically displays these data.
