# Supplementary material for: Simplified, Physically Motivated, and Broadly Applicable Range-Separation Tuning
Source: J Phys Chem Lett. 2025 Aug 4;16(32):8198–208. doi: 10.1021/acs.jpclett.5c01441 (PMC12359113; doi:10.1021/acs.jpclett.5c01441)
Supplement: Supplementary file 2 [file jz5c01441_si_002.pdf]

jz-2025-01441p.R1

Name: Peer Review Information for "Simplified, Physically Motivated, and Broadly Applicable Range-Separation Tuning"

First Round of Reviewer Comments

Reviewer: 1

Comments to the Author

In the manuscript by Smiga and co-workers, a new approach is presented for the system-dependent tuning of the range-separation parameter in range-separated hybrid density functionals. The results for molecular systems are quite promising. The manuscript definitely deserves publication provided that some concerns are addressed and its technical quality is improved.

The major problem of the approach presented in the manuscript is that the range-separation parameter is evaluated from densities averaged over the entire system (or unit cell for solids). While it is not problematic for solids, it causes the violation of size consistency for molecular systems. This aspect should be discussed in the manuscript.

Eq. 1: pls. introduce omega.

Page 5, line 8: ... epsilon^LSDA_xc ...

Page 6: it is not clear how n_th was chosen and what its value is. Was it determined on the basis of Fig. S1? There, r_c does not reach saturation.

Page 6: the source of the geometries of the test systems should be given.

Page 9: pls. explain what omega_eff, omega_IE, and omega_0.4 mean.

Page 15, last line: what is N? What tuning is meant here?

Page 16: what is BNL?

Page 16: the cc-pVDZ basis used for the photo-voltaic systems is rather small, especially if the results are compared to experimental ones. For that purpose, a larger basis set, e.q., aug-cc-pVTZ, should be used.

Table 3: the caption of the table is vague. It is not clear what is presented in the table. How are the mean errors calculated?

Fig. 5: what is the reason for the jump at n=10 for the acenes?

Pages 17-18, paragraph "For the (PPV)n = ...": looking at fig. 5, it seems that the statements for the PPV and (p-phenyl)nitroaniline oligomers have been transposed.

Tables 4 and 5 and their discussion cannot be followed. In particular, all the notations and acronyms should be introduced. The solid-state physics jargon should be avoided (e.g., what does vacuum size mean?). The significance of these numerical experiments should be clearly explained.

Typos:

Caption of Fig. 4: photo-oltaic -> photo-voltaic

Caption of Fig. 5: ... in Tables SI5, ...

Page 17, line 33: ... distinct cutoff introduced in ...

Page 17, line 35: … which it is related to.

Table 5, caption: Bracket -> Bracketed

Page 20, line 13: predicate is missing.

References: there are several typos, which should be fixed, and a uniform citation format should be used.

Reviewer: 2

Comments to the Author

1. What is the major advance reported in the paper?

Smiga and coworkers report the development of a nonempirical approach to determine the range-separation parameter of range-separated hybrid functionals. As an alternative to the optimal tuning procedure, their method is also system-dependent but significantly less computationally demanding. The range-separation parameter is calculated on the fly from the electron density of the system.

The accuracy of the approach is tested for computing charge-transfer and Rydberg excitations of molecular systems, as well as ionization potentials and optical gaps of organic photovoltaics. Preliminary insights are also provided for applying the method to solid-state systems, which could represent a major advance in the field.

2. What is the immediate significance of this advance?

The research reported here can be considered as an interesting development in DFT that can easily broaden the use of system dependent range-separated hybrids to better predict electronic properties of extended systems.

3. Technical suggestions

The paper is very well written and, in my opinion, could merit publication in The Journal of Physical Chemistry Letters. Prior to possible publication, I would like to share the following comments:

i) Page 3, line 19: The authors state that none of the existing approaches to determine the range-separation parameter is universal. This also applies to the method developed in the present work. In Refs. https://doi.org/10.1063/5.0010976 and https://doi.org/10.1063/1.5097164, it is shown that a value between 0.4 and 0.5 a.u. offers a reasonable statistical compromise. It would be valuable for the authors to comment on this, particularly to clarify why LC-wPBE with $\omega = 0.4$ was chosen as a benchmark.

ii) From Table 1, the average values of wIE and weff can be estimated to be approximately 0.31 and 0.27 a.u., respectively. For the sake of comparison, it might be beneficial to include excitation energies computed with LC-wPBE using w = 0.3 a.u., to provide readers with a more complete picture.

iii) It is well known that long-range corrected hybrid functionals are generally not recommended for solids, as the exact-exchange term decays more slowly than the DFT exchange and does not cancel out efficiently. This is one of the motivations behind the development of functionals such as HSE for solid-state applications. The authors could expand on why their approach might still be promising in comparison to HSE, particularly in light of this limitation.

iv) In the Conclusions, the authors state: "This is particularly important for modeling excitations in molecules, clusters, and solids." This claim should be moderated, as the method has not yet been fully validated for solid-state systems.

Reviewer: 3

Comments to the Author

The authors report on a range-separated hybrid where the range-separation parameter is computed directly from the electron density via a scheme based on the LDA kernel.

In principle this is an interesting study, but I have several significant concerns that I would like to see addressed before I can make a final recommendation:

1. The authors argue that a major advantage of their scheme is that multiple calculations, as in IE tuning, are not necessary. However, which density do they base their calculation on? And is this density self-consistent with the one obtained from the range-separation parameter? If not, what's the effect of self-consistency?

2. It is unclear how many adjustable parameters the authors employ. For molecules, what's n_th and how is it determined? How is epsilon computed in solids? Are there other empirical parameters in the process?

3. In Table 4 for the bulk materials - no gaps are given. How do we know if the result is good? Also, are 2d and 3d materials screened in the same way? And why the choice of n_c = n_th = 6.96x10^-4 e/bohr?

4. Especially based on the scant evidence for solids, I think that the "universally applicable" in the title is premature.

5. The claim that standard tuning is difficult to extend to solids is incorrect - see the article's Ref. 42 for an extension based on Wannier functions.

6. The extension to Wannier functions is also necessary for longer molecular chains - see Ohad et al. JCTC 20, 7168 (2024). Therefore the comparison to standard tuning is problematic because the standard tuning is not good. Also very long acenes do not make sense - they have never been synthesized and there's a debate whether they would really be of a singlet ground state nature.

7. What are the numbers in parentheses in Figure 2?

Author's Response to Peer Review Comments:

Author’s Response to Reviews of

Simplified, Physically Motivated, and Broadly Applicable Range-Separation Tuning

Aditi Singh, Subrata Jana, Lucian A. Constantin, Fabio Della Sala, Szymon Smiga´

The Journal of Physical Chemistry Letters

Manuscript ID: jz-2025-01441p

RC: *Reviewer’s Comment*, AR: *Author’s Response*

Dear Editor,

Thank you for your communication on June 5th, 2025. Please find our response to the Referee’s reports and a summary of the changes made to the manuscript. In the revised manuscript, we considered all the suggestions pointed out by the Referees.

With two favorable referee reports recommending publication without further revisions, we hope the manuscript gains your approval for publication in The Journal of Physical Chemistry Letters (ACS).

Sincerely,
Szymon Smiga (for all the authors)´

We thank all Referees for reviewing our manuscript and for valuable suggestions and comments. We respond point by point to all comments. Changes done in the present form of the manuscript are indicated in the color format.

Major changes in the manuscript

- We have added the Table of Contents graphic with appropriate dimensions.
- We have corrected the reference formatting.
- We have corrected all the typos pointed out by the referee.

Reviewer #1

RC: The major problem of the approach presented in the manuscript is that the range-separation parameter is evaluated from densities averaged over the entire system (or unit cell for solids). While it is not problematic for solids, it causes the violation of size consistency for molecular systems. This aspect should be discussed in the manuscript.

AR: *We acknowledge the Referee's point. However, conventional tuned range-separated hybrids inherently lack size-consistency, as established in Ref. [https://doi.org/10.1063/1.4807325]. Our primary aim here is to introduce a simplified, density-reliant ω tuning scheme with broad applicability. We have addressed this scope explicitly in the revised manuscript.*

RC: Eq. 1: pls. Introduce omega.

AR: *We appreciate the Referee's helpful observation. The tuned range-separation parameter ω is now clearly defined and emphasized in the revised manuscript.*

RC: Page 5, line 8: ...ϵ_{xc}^{LSDA} ...

AR: *We are grateful for this clarification.*

RC: Page 6: it is not clear how n_{th} was chosen and what its value is. Was it determined on the basis of Fig. S1? There, r_c does not reach saturation.

AR: *We thank the reviewer for this valuable suggestion, which has been addressed in the revised manuscript. The parameter n_{th} =0.0164 was specifically optimized to define a cutoff radius ensuring consistent ω values across charge-transfer molecules via IE tuning. We have updated Fig S1 to include a plot justifying our selection of n_{th}. Fig S1 demonstrates the close agreement between ω_{eff} and ω_{IE} for linear acenes (n =2-40), poly(p-phenylenevinylene) molecules [$(PPV)_{n=1-8}$], and poly(p-phenyl)nitroaniline [$O_2N(Ph)_{n=1-11}NH_2$] oligomers. (see modified Fig S1).*

RC: Page 6: The source of the geometries of the test systems should be given.

AR: *We appreciate your comments. This has now been addressed.*

RC: Page 9: pls. explain what ω_{eff}, ω_{IE}, and $\omega_{0.4}$ mean.

AR: *Thank you for pointing this out. This work employs:* ω_{eff} *(newly tuned parameter),* ω_{IE} *(ionization-energytuned parameter), and* $\omega_{0.4}$ *(fixed* $\omega = 0.4$ *reference). This is now defined throughout the text.*

RC: Page 15, last line: what is N? What tuning is meant here?

AR: *Here* N *stands for HOMO and* $N+1$ *denotes the LUMO orbital. And the tuned range separated parameter is optimized based on both HOMO and LUMO orbital energies. The manuscript has been revised accordingly.*

RC: Page 16: what is BNL?

AR: *Here BNL stands for Baer, Neuhauser, and Livshits. We have addressed this.*

RC: Page 16: the cc-pVDZ basis used for the photo-voltaic systems is rather small, especially if the results are compared to experimental ones. For that purpose, a larger basis set, e.q., aug-cc-pVTZ, should be used.

AR: *Your concerns are correct with regards to smaller basis like cc-pVDZ, but to maintain the consistency between the original work and our method, we used a similar basis as mentioned in the paper. But, in the SI4, we present the result for a bigger basis, i.e., def2-QZVP, alongside the original one. We observe minimal overall impact, with the MAE exhibiting only marginal variation.*

RC: Table 3: The caption of the table is vague. It is not clear what is presented in the table. How are the mean errors calculated?

AR: *For consistency with Ref. [10.1021/acs.jpclett.5b00086], we recalculated the error metric as the mean absolute error (MAE) rather than the mean error. All values are benchmarked against experimental data from Ref. [10.1002/cphc.200390047]. This clarification has been incorporated into the revised manuscript.* RC: Fig. 5: what is the reason for the jump at n=10 for the acenes?

AR: *A discontinuity is evident when comparing acenes with n=10 and n=11 rings, attributable to a sudden change in the Kohn-Sham gap. This observation implies the emergence of an open-shell biradicaloid singlet ground state in longer acenes. This has been reported here https://doi.org/10.1021/acs.jpclett.5c00086. We have also discussed it briefly in our revised manuscript.*

RC: Pages 17-18, paragraph "For the (PPV)n = ...": looking at fig. 5, it seems that the statements for the PPV and (p-phenyl)nitroaniline oligomers have been transposed.

AR: *We appreciate the reviewer's comprehensive analysis. It is now corrected.*

RC: Tables 4 and 5 and their discussion cannot be followed. In particular, all the notations and acronyms should be introduced. The solid-state physics jargon should be avoided (e.g., what does vacuum size mean?). The significance of these numerical experiments should be clearly explained.

AR: *We commend the reviewer's insightful clarification. Within periodic computational frameworks, 'vacuum size' defines the engineered empty-space dimension isolating structures (surfaces, slabs, etc.), crucially governing boundary-condition implementations. The manuscript has been revised to include this specification.*

RC: Typos: Caption of Fig. 4: photo-oltaic -> photo-voltaic Caption of Fig. 5: ... in Tables SI5, ... Page 17, line 33: ... distinct cutoff introduced in ... Page 17, line 35: ... which it is related to. Table 5, caption:

Bracket -> Bracketed Page 20, line 13: predicate is missing. References: there are several typos, which should be fixed, and a uniform citation format should be used.

AR: *Thank you for pointing this out. Now it is corrected.*

Reviewer #2

RC: Page 3, line 19: The authors state that none of the existing approaches to determine the rangeseparation parameter is universal. This also applies to the method developed in the present work. In Refs. https://doi.org/10.1063/5.0010976 and https://doi.org/10.1063/1.5097164, it is shown that a value between 0.4 and 0.5 a.u. offers a reasonable statistical compromise. It would be valuable for the authors to comment on this, particularly to clarify why LC-wPBE with = 0.4 was chosen as a benchmark.

AR: *We are grateful to the reviewer for posing this insightful question. We note that the primary difference between finite-size systems and bulk solids concerns how the average* $\langle r_s \rangle$ *is determined. Nevertheless, we wish to emphasize that our scheme remains valid for both. As the reviewer notes, and consistent with studies such as Refs. https://doi.org/10.1063/5.0010976 and https://doi.org/10.1063/1.5097164,values of* ω *between 0.4 and 0.5* $Bohr^{-1}$ *are recognized as offering a reasonable statistical compromise for many properties across diverse systems. The established value of* ω =0.4 *for the LC-wPBE benchmark specifically because it is a standardized, well-documented reference point resulting from rigorous optimization studies https://doi.org/10.1063/5.0010976. This optimization aimed to balance accuracy across key properties like thermochemistry, barrier heights of chemical reactions, bond lengths, and long-range charge transfer.*

We like to draw the attention towards the study [https://doi.org/10.1021/acs.jctc.5b01144], that ω = $0.4 Bohr^{-1}$ *is not universally optimal. Performance can degrade for systems involving strong non-covalent interactions, transition metal chemistry, specific reaction barriers, or highly conjugated systems – precisely the type of limitations that motivate the development of system-dependent approaches like ours. The choice of this benchmark underscores the need for, and potential advantage of, methods that can adapt the range-separation parameter to the specific electronic environment.*

RC: From Table 1, the average values of wIE and weff can be estimated to be approximately 0.31 and 0.27 a.u., respectively. For the sake of comparison, it might be beneficial to include excitation energies computed with LC-wPBE using w = 0.3 a.u., to provide readers with a more complete picture.

AR: *We thank the reviewer for noting this - the requested data are now included in the SI. The mean absolute error for LC-wPBE using w = 0.3 a.u. is 0.19 eV comparable to the GDD scheme, whereas* ω_{eff} *continues to deliver the best performance overall.*

RC: It is well known that long-range corrected hybrid functionals are generally not recommended for solids, as the exact-exchange term decays more slowly than the DFT exchange and does not cancel out efficiently. This is one of the motivations behind the development of functionals such as HSE for solid-state applications. The authors could expand on why their approach might still be promising in comparison to HSE, particularly in light of this limitation.

AR: *This is an interesting question. As the referee correctly pointed out, HSE06 is indeed a widely used and effective hybrid functional for solids. However, it is known to underestimate band gaps in insulators. This limitation has been addressed by dielectric-dependent hybrid (DDH) functionals, where the long-range exchange term is modified to decay as* $V_x^{LR} \sim \frac{1}{\varepsilon r}$*, where* ε *is the dielectric constant. This method works well for both the semiconductors and insulators. A further development involves range-separated dielectricdependent hybrids, which require the determination of an additional parameter,* μ*, commonly referred to as the screening parameter (in our case is denoted as* ω*). Various strategies have been proposed in the literature to construct* μ*. In this manuscript, we argue that our proposed definition of* μ *is general and can be reliably employed as a screening parameter in screened dielectric-dependent hybrid functionals.*

RC: In the Conclusions, the authors state: "This is particularly important for modeling excitations in molecules, clusters, and solids." This claim should be moderated, as the method has not yet been fully validated for solid-state systems.

AR: *We accept your recommendation. Thus, have modified the statement.*

Reviewer #3

RC: The authors argue that a major advantage of their scheme is that multiple calculations, as in IE tuning, are not necessary. However, which density do they base their calculation on? And is this density self-consistent with the one obtained from the range-separation parameter? If not, what's the effect of self-consistency?

AR: *Thank you for this valuable question. The basis sets used for all charge-transfer and other electronic structure calculations presented in this work are specified either in the figure captions or in the Supporting Information. Regarding the evaluation of the range-separation parameter ω, we emphasize that it is largely independent of the density and/or basis set. In our procedure, we first perform standard DFT calculations using the PBE exchange-correlation (XC) functional. The resulting PBE density is then used to evaluate ω. This is the protocol consistently followed throughout this work. While it is indeed possible to evaluate ω "on the fly" during self-consistent calculations, such an implementation is not adopted here, as the impact of this approximation is expected to be minimal. For example, in the GDD approach of ref.[doi:10.1021/jp405827x], the parameter ω_{GDD} is also determined from a single evaluation using a fixed density, as discussed after Eq. (15) in that reference. We also discuss it in the main text.*

RC: It is unclear how many adjustable parameters the authors employ. For molecules, what's n_{th} and how is it determined? How is epsilon computed in solids? Are there other empirical parameters in the process?

AR: *We value these thoughtful questions and respond point-by-point below:*

(i) It is unclear how many adjustable parameters the authors employ - We only have one adjustable parameteri.e., n_{th}

(ii) For molecules, what's n_{th} and how is it determined? - Our choice of threshold density n_{th} =1.64×10^{-2} e/bohr^3 is based on its ability to produce closely matching ω values for the majority of charge-transfer molecules obtained via IE tuning. For further clarification, please refer to the revised manuscript and the Fig S1 from supporting Information.

(iii) How is epsilon computed in solids - The ϵ values for molecular crystal calculations are already available from the supporting information of ref. Journal of Chemical Theory and Computation 2018, 14, 2919–2929. This ϵ was used for OT-SRSH, and in our present case, we use the same ϵ. This has been added to the caption for Table 5.

(iv) Are there other empirical parameters in the process? - No, there are no other empirical parameter in theconstruction of the ω_{eff}.

RC: In Table 4 for the bulk materials - no gaps are given. How do we know if the result is good? Also, are 2d and 3d materials screened in the same way? And why the choice of n_c = n_{th} = $6.96\text{x}10^{-4}$ e/bohr?

AR: *Thanks for raising this concern. We would comment on it pointwise:*

A) In Table 4 for the bulk materials - no gaps are given - We would like to comment that for bulk systems, onerequires a dielectric-dependent hybrid instead of a long-range hybrid as assessed in this work. The dielectricdependent hybrid is well established in various papers (i) https://doi.org/10.1103/PhysRevMaterials.2.073803 (ii) https://doi.org/10.1021/acs.jpclett.8b00919 (iii) https://doi.org/10.1103/PhysRevB.108.045101.

B) How do we know if the result is good? - The bulk systems, the performance of the dielectric-dependenthybrid is well studied in different previous literature with different μ values considered in the work. See refs https://doi.org/10.1103/PhysRevMaterials.2.073803 and (iii) https://doi.org/10.1103/PhysRevB.108.045101. Also, note that for bulk systems the present ω_{eff} is not changing compared to μ_{eff}^{fit}. So, there will be no change in band gaps as already assessed in https://doi.org/10.1103/PhysRevB.108.045101

C) Also, are 2d and 3d materials screened in the same way? - For monolayers and surfaces, the definition ε (dielectric constant) is not well established. It was only addressed recently in ref. https://doi.org/10.1103/8vvnk9p3 and arXiv:2505.17261. It will be interesting to apply the present scheme for monolayers, but that requires a details study and it is a matter for another paper.

D) And why the choice of n_c = n_{th} = 6.96x10^{-4} e/bohr? - We would like to refer to this paper ref. https://doi.org/10.1103/PhysRevB.108.045101. For bulk systems and 2D systems n_{th} = 6.96x10^{-4} e/bohr^3 is proposed in ref. J. Chem. Phys. 155, 104103 (2021) and Phys. Rev. Rev. B 101, 245163 (2020). We do not want to change those for bulk systems as already mentioned in paper.

RC: Especially based on the scant evidence for solids, I think that the "universally applicable" in the title is premature.

AR: *We appreciate the reviewer's valuable comment. While the implementation of the averaging scheme may differ between bulk solids and finite systems due to their distinct treatments, the core methodology remains universally valid. We plan to further investigate these variations in forthcoming work. Regarding the paper's title, we changed to "broadly applicable".*

RC: The claim that standard tuning is difficult to extend to solids is incorrect - see the article's Ref. 42 for an extension based on Wannier functions.

AR: *We acknowledge the referee's valuable suggestion. While this approach is conceptually viable, we cannot currently assess the computational feasibility of implementing a 'Wannier-tuned' hybrid functional, as these methods are publicly unavailable. Relevant methodological foundations are appropriately cited in our work, [https://doi.org/10.1073/pnas.2104556118 and https://doi.org/10.1021/acs.jctc.4c00847].*

RC: The extension to Wannier functions is also necessary for longer molecular chains - see Ohad et al. JCTC 20, 7168 (2024). Therefore the comparison to standard tuning is problematic because the standard tuning is not good. Also very long acenes do not make sense - they have never been synthesized and there's a debate whether they would really be of a singlet ground state nature.

AR: *We acknowledge this comment. Without engaging in methodological debate, we would instead like to cite the relevant work [https://doi.org/10.1021/acs.jctc.4c00847].*

RC: What are the numbers in parentheses in Figure 2?

AR: *Thank you for highlighting this. The values in parentheses denote ω_{eff} for each respective atom. We have added this clarification to the caption, and the manuscript has been revised.*
